# Exploring fluoropolymers for fabrication of femtoliter chamber arrays used in digital bioanalysis

**DOI:** 10.1038/s41598-024-61726-8

**Published:** 2024-05-20

**Authors:** Jun Ando, Kazue Murai, Makiko Mori, Tomoe Michiyuki, Tatsuya Iida, Asami Makino, Hajime Shinoda, Rikiya Watanabe

**Affiliations:** Molecular Physiology Laboratory, Cluster for Pioneering Research, RIKEN, Saitama, Japan

**Keywords:** Biotechnology, Nanobiotechnology

## Abstract

The global supply of fluoropolymers and fluorinated solvents is decreasing due to environmental concerns regarding polyfluoroalkyl substances. CYTOP has been used for decades primarily as a component of a femtoliter chamber array for digital bioanalysis; however, its supply has recently become scarce, increasing the urgency of fabricating a femtoliter chamber array using alternative materials. In this study, we investigated the feasibility of fabricating a femtoliter chamber array using four types of fluoropolymers in stable supply as candidate substitutes and verified their applicability for digital bioanalysis. Among these candidates, Fluorine Sealant emerged as a viable option for fabricating femtoliter chamber arrays using a conventional photolithography process. To validate its efficacy, we performed various digital bioanalysis using FP-A-based chamber arrays with model enzymes such as CRISPR–Cas, horseradish peroxidase, and β-galactosidase. The results demonstrated the similar performance to that of CYTOP, highlighting the broader utility of FP-A in digital bioanalysis. Our findings underscore the potential of FP-A to enhance the versatility of digital bioanalysis and foster the ongoing advancement of innovative diagnostic technologies.

## Introduction

Digital bioanalysis using femtoliter chamber arrays has emerged as a transformative tool that plays a pivotal role in advancing several areas of life sciences^1,2^. This innovative approach has demonstrated a significant impact on single molecule biophysics, providing insights into biomolecules with unprecedented sensitivity and precision^3–8^. Additionally, the impact of digital bioanalysis is reverberating in the field of disease diagnostics, and the technique has emerged as an invaluable testing platform for infectious diseases and latent disorders^5,6^. Its innate capacity for high sensitivity and precision enables the detection of even the rarest biomarkers or pathogens, thereby facilitating early disease diagnosis and continuous disease monitoring. The versatility of digital bioanalysis underscores its importance in advancing both basic research and medical applications.

Femtoliter chamber arrays for digital bioanalysis can be classified into two main types: (i) those fabricated by photolithography of fluoropolymers^9^ and (ii) those fabricated by injection molding of plastics such as cyclo-olefin polymer (COP)^10^ and polycarbonate (PC)^11,12^. In terms of the cost of manufacturing equipment and well-established manufacturing processes, femtoliter chamber arrays made of fluoropolymers have been widely used in basic research for decades^2,13^. Recently, the global supply of fluoropolymers and fluorinated solvents has decreased because of environmental concerns regarding polyfluoroalkyl substances (PFAS)^14^. CYTOP has historically played a central role as a component of femtoliter chamber arrays^2,13^; however, recent scarcity in the supply chain has heightened the necessity of exploring alternative materials for fabricating femtoliter chamber arrays. This exploration could contribute significantly to the sustainability of research and development in digital bioanalysis.

In this study, we investigated the feasibility of fabricating a femtoliter chamber array using four types of fluoropolymers with a stable supply. Successfully fabricated femtoliter chamber arrays were used to verify the feasibility of digital bioanalysis, demonstrating a performance similar to that of conventional CYTOP. Thus, it is applicable as an alternative to digital bioanalysis.

## Results

### Formation of a fluoropolymer film

**Figure 1:**
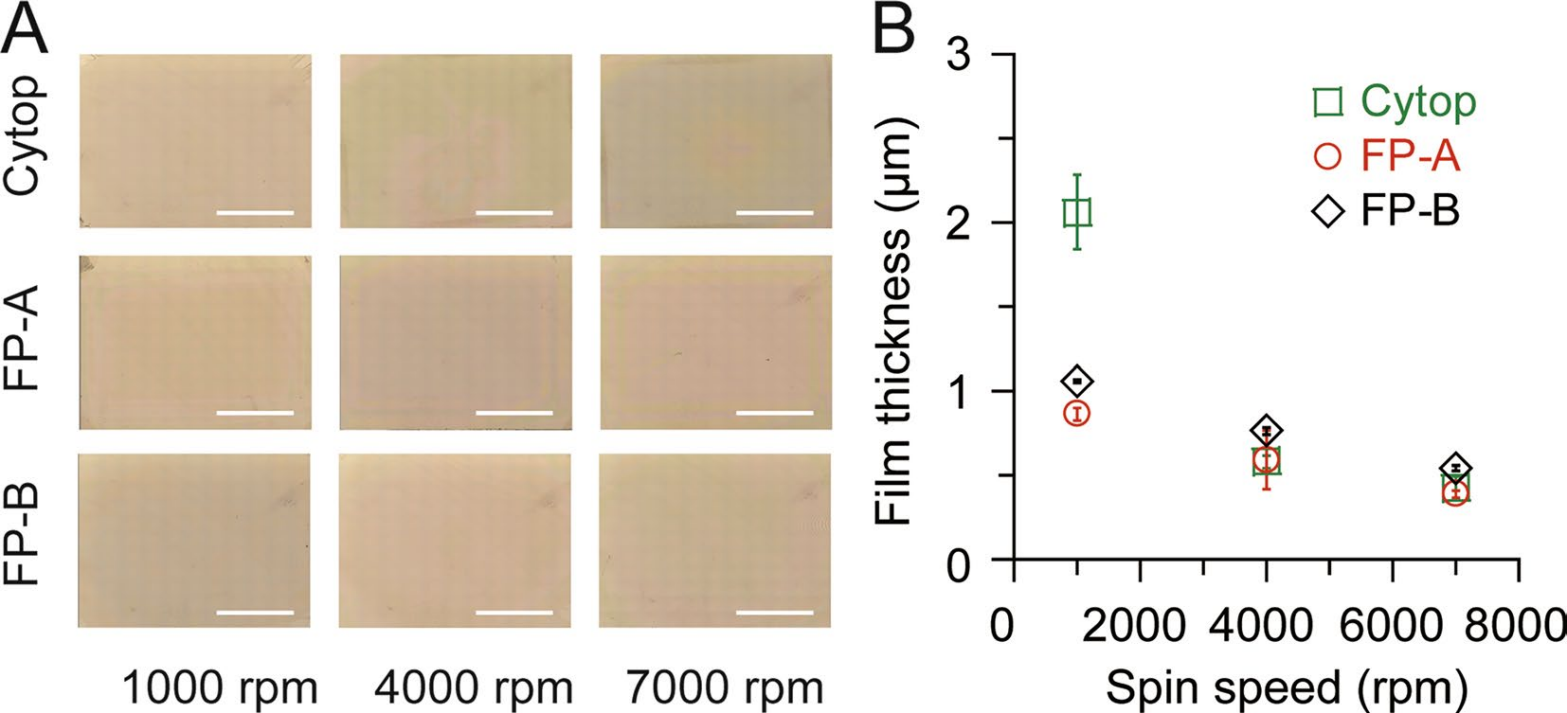
Spin-coating of fluoropolymer on glass substrate. (**A**) Photographs of spin-coated glass substrate. Scale bar represent 10 mm. (**B**) Thickness of fluoropolymer film plotted against spin speed. Green, red, and black represent CYTOP, FP-A, and FP-B, respectively (n = 3 replicates).

Various methods are used for the formation of thin polymer films, including spin-coating, dip-coating, spray-coating, and bar-coating. In this study, thin films were formed on glass substrates using four types of fluoropolymers as alternatives to CYTOP by spin coating, which is a widely utilized technique for fabricating femtoliter chamber arrays (Fig. 1). Four types of fluoropolymers, FP-A (Fluorine Sealant DKA-001, DAIKIN INDUSTRIES), FP-B (SFCOAT SFE-GP03JL, AGC seimi chemical), FP-C (SURECO 2101S, AGC), and FP-D (SURECO 2120X, AGC), are commercially available that are mainly used for moisture-proof coatings of electronic substrates. Because a high water-contact-angle is preferred as a component of a femtoliter chamber array, we selected the fluoropolymers with contact angles above 110° (FP-A: 115°, FP-B: 117°, FP-C and D: 113°), which is equivalent to that of conventional CYTOP (112°).

FP-A and FP-B were able to form thin films with a uniform thickness between 0.4 and 1.1 µm on glass substrates by spin coating (Fig. 1), whereas FP-C and FP-D failed owing to a low concentration of fluoropolymer or low substrate affinity. The film thicknesses of FP-A and FP-B decreased with increasing spin speed, indicating that the film thickness was controlled by the spin-speed. At a low spin speed of 1000 rpm, the film thickness of CYTOP was twice those of FP-A and FP-B. A possible reason for this discrepancy could be the high boiling point of the solvent of CYTOP (Ct-Solv180, 174 °C) compared to that of FP-A (Novec 7200, 76 °C) and FP-B (mixture, 127 °C), which could affect the viscosity during spin-coating.

### Fabrication of femtoliter chamber arrays

**Figure 2:**
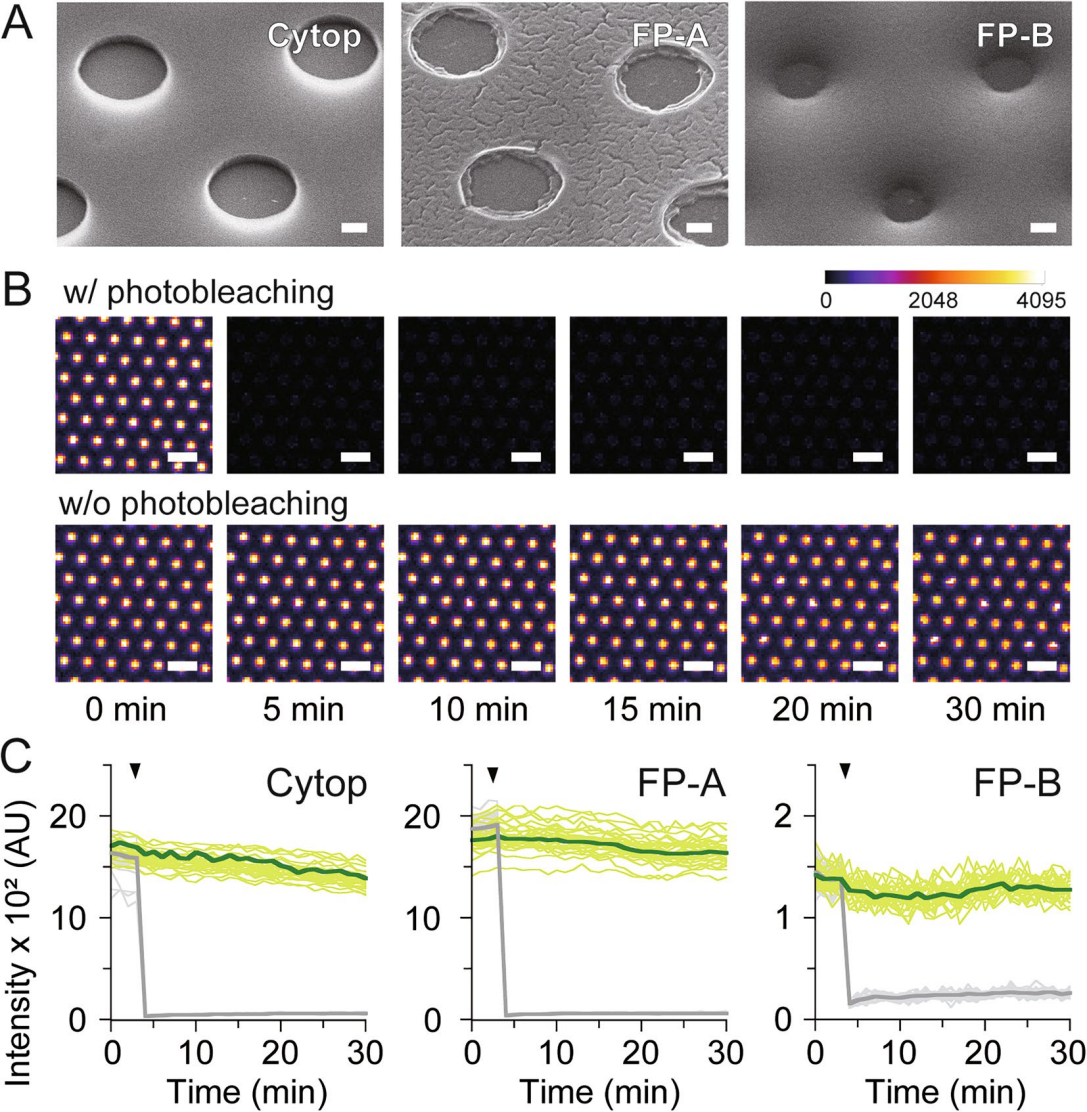
Femtoliter chamber arrays. (**A**) SEM images of femtoliter chambers fabricated with CYTOP, FP-A and FP-B films, respectively. Scale bars represent 1 μm. (**B**) Fluorescence images of FP-A chambers filled with 10 µM Alexa 488 during the FRAP assay. Scale bars represent 10 µm. (**C**) Time course of fluorescence intensity of Alexa 488 with (light grey and grey represent 35 chambers and their average, respectively) or without photobleaching (light green and green represent 35 chambers and their average, respectively). Black allows indicate the timing of photobleaching.

Using conventional photolithography, we fabricated femtoliter-volume chamber arrays made of fluoropolymers (FP-A and FP-B) that can be used to form uniform thin films as described above (Figs. 2A, S1, and S2). Femtoliter chamber arrays can be fabricated with the fluoropolymers via a photolithographic process similar to that previously reported for CYTOP with chamber volumes of 2.8–6.7 fL (Fig. S3); however, some problems were encountered with each fluoropolymer. For FP-A, if the solvent was not sufficiently removed after spin-coating, the film shrank when exposed to vacuum conditions in the RIE machine, resulting in cracks in the deposited photoresist film on top of the FP-A (Fig. S4). Therefore, it was necessary to sufficiently remove the solvent by heating the substrate at 70 °C for more than 12 h. Film formation of the photoresist was another challenge owing to the poor adhesion to FP-A after prolonged baking. To improve adhesion to FP-A, a fluorinated surfactant (Surflon S386) was added to the photoresist to form a thin film. In addition, during dry-etching, the mesh structure remained on the bottom surface of the glass regardless of the processing time (Fig. S5). Therefore, ultrasonic treatment with a strongly alkaline solution after dry-etching was necessary to remove the mesh structure and form the uniform through-hole structures. In contrast, for FP-B, numerous small protrusions occurred on the surface of the membrane, producing approximately 0.3% defects (Fig. S6).

For digital bioanalysis, the reaction solution was dropped into an array of chambers and then replaced with oil to fractionate and retain the solution in each chamber. Because the stable retention of the reaction solution depends on the composition of the chamber and the type of oil, femtoliter chamber arrays with similar volumes (V: 4–5 fL) were fabricated using CYTOP, FP-A, and FP-B, to investigate the retention times of the reaction solutions in each chamber (Fig. 2B,C). When an aqueous solution containing a green fluorescent dye (Alexa 488), was used as an indicator and fractionated into chambers using Fomblin oil, which is commonly used in digital bioanalysis, the fluorescence intensity of the chambers remained stable for longer than 30 min regardless of the type of fluoropolymer (Fig. 2B,C), indicating the stable retention of solutes in the chambers. In addition, a fluorescence recovery after photobleaching (FRAP) assay was performed to ensure that the chambers were hermetically sealed and not conducting. The femtoliter chambers were filled with a solution containing 10 µM Alexa 488, and some of them were selectively photobleached using the laser scanning system of a confocal microscope (Fig. 2B,C). Subsequent analysis of fluorescence intensity showed that the bleached chamber did not recover its fluorescence in ~ 30 min, confirming that the chamber was hermetically sealed with Fomblin oil.

### Fluorescence properties

**Figure 3:**
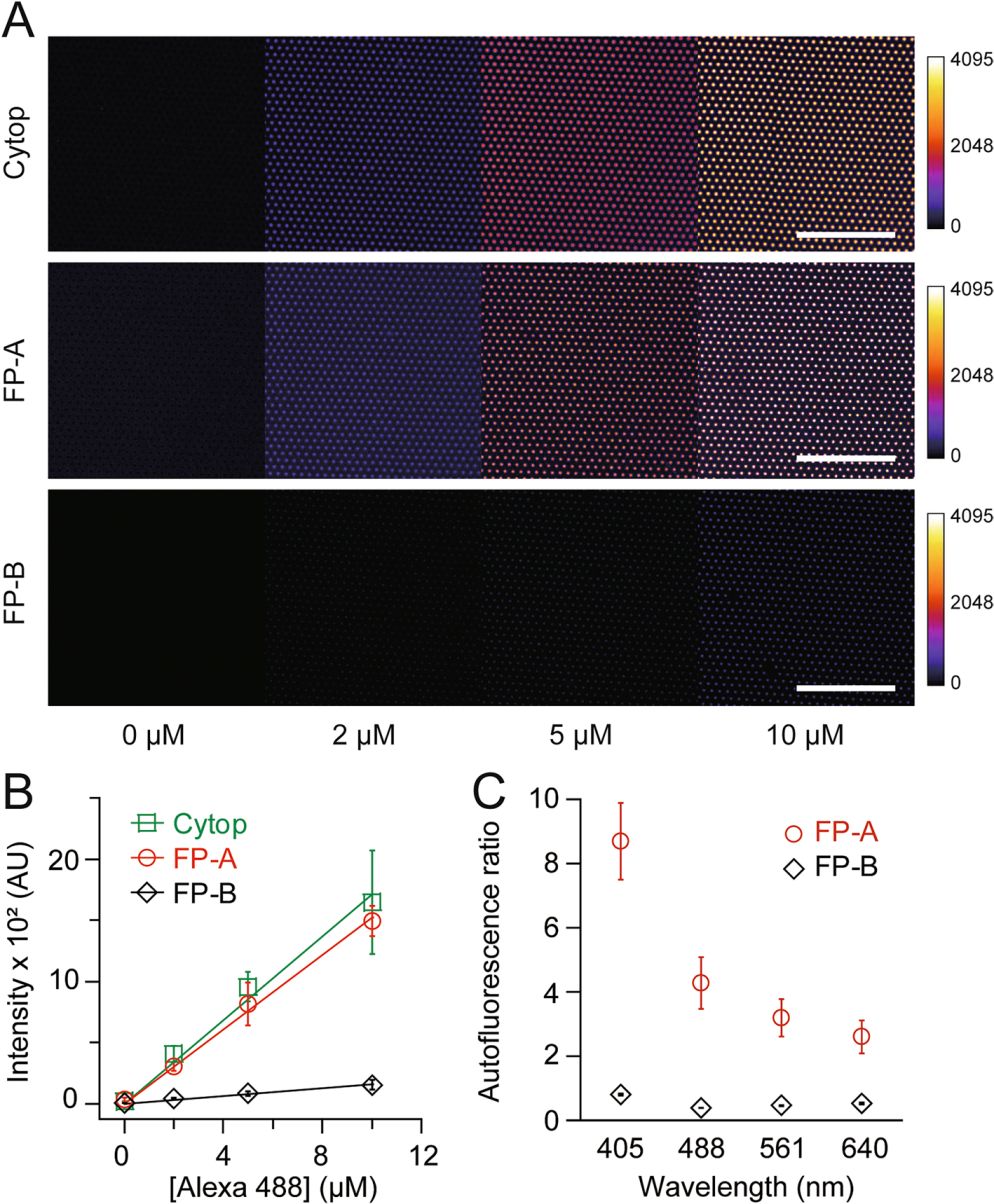
Fluorescence imaging. (**A**) Representative fluorescence images and (**B**) fluorescence intensities of femtoliter chamber arrays prepared using CYTOP, FP-A or FP-B with an indicated concentration of Alexa 488. Solid lines show linear regressions. Scale bars represent 100 µm. (**C**) Ratio of autofluorescence intensity to CYTOP (n = 3 technical replicates).

The efficiency of encapsulation of the fluorescent dye solution in the chambers can affect the reproducibility and quantitation of digital bioanalysis. To investigate the efficiency, the fluorescence intensity was measured using 4–5 fL chamber arrays, encapsulated with a solution containing the indicated concentration of Alexa 488 (Fig. 3A). The fluorescence intensity increased proportionally with the concentration of Alexa 488, regardless of the type of fluoropolymer. However, the fluorescence intensity of FP-A was comparable to that of CYTOP, whereas that of FP-B was approximately one tenth (Fig. 3B). The lower fluorescence intensity in FP-B may be attributed to the open-front structure of the chamber (Fig. 2A), which may allow oil to enter the chamber through the opening, resulting in a low actual volume of the dye solution in the chamber.

The autofluorescence of fluoropolymers can also affect fluorescence imaging, resulting in lower sensitivity of digital bioanalysis. Autofluorescence was measured using four laser light sources widely used in digital bioanalysis: 405, 488, 561, and 640 nm (Fig. 3C). FP-B showed a similar or lower autofluorescence intensity than CYTOP at all wavelengths, whereas FP-A showed a higher, especially at shorter wavelengths. However, the autofluorescence for all fluoropolymers, even for FP-A, was almost negligible compared to the signal from the fluorescent dyes (Fig. 3A,B), demonstrating the feasibility of digital bioanalysis with new fluoropolymers. Collectively, FP-A has the potential to be used as an alternative to CYTOP for digital bioanalysis owing to its fluorescence properties.

### Demonstration of digital bioanalysis

Digital bioanalysis is an exceptionally sensitive approach that can fractionate target biomolecules of interest at the single-molecule level for subsequent detection by coupling to the fluorogenic reaction of enzymes^1,2^. The detection modalities of digital bioanalysis can be broadly classified as (i) direct detection of target enzymes and (ii) indirect detection of target molecules by fluorogenic enzymes^15^.

**Figure 4:**
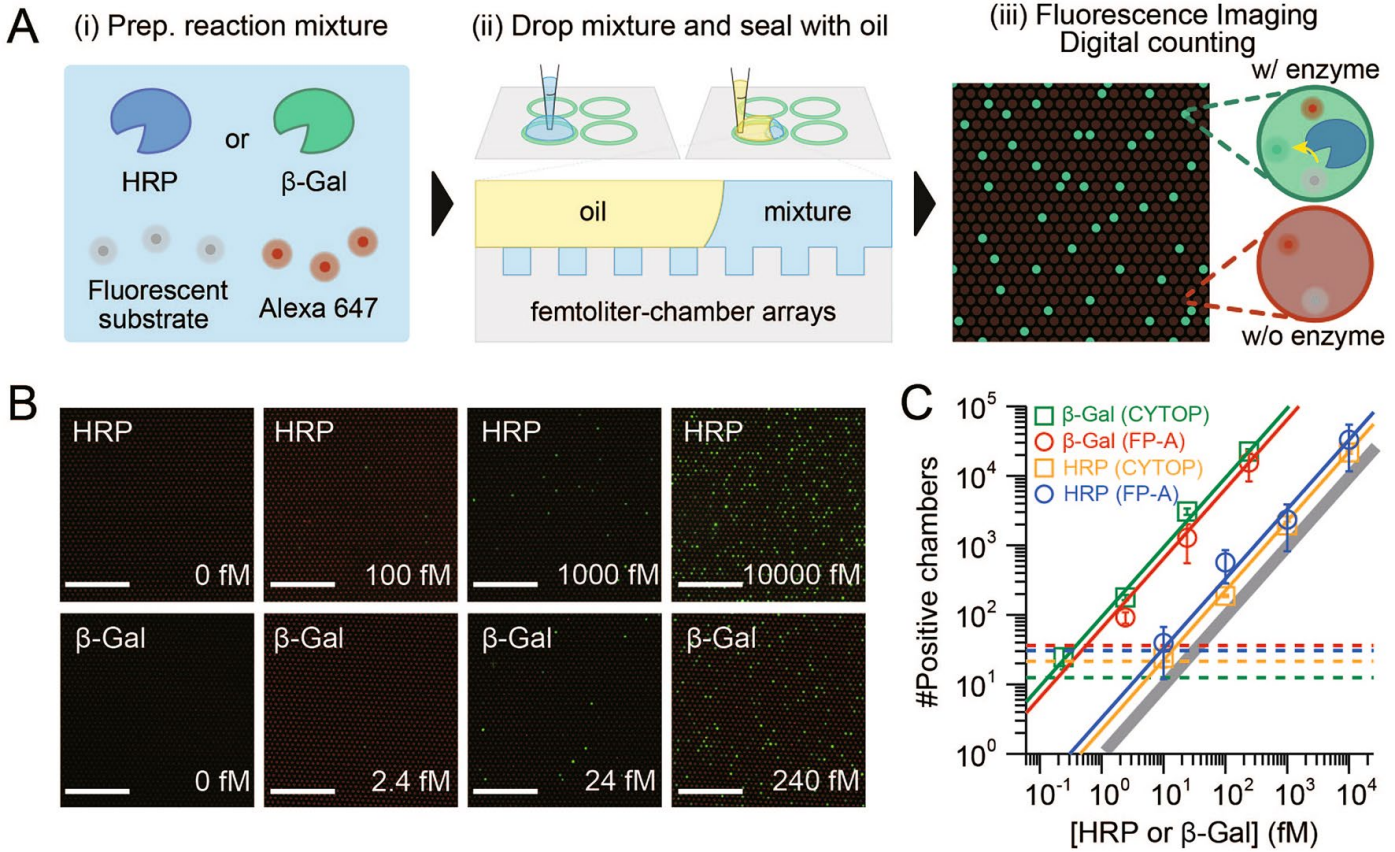
Digital enzyme detection of HPR and β-Gal. (**A**) Schematics of digital enzyme detection assay. (**B**) Representative fluorescence images at each concentration of enzyme using FP-A. Scale bars represent 100 µm. (**C**) Number of positive chambers obtained using β-Gal_CYTOP (green), β-Gal_FP-A (red), HRP_CYTOP (yellow), or HRP_FP-A (blue). The solid lines show the linear regressions in red, green, yellow, and red and the theoretical value in grey. The dotted lines show the values of the blank mean + 3 SD (n = 3 technical replicates).

Direct detection of target enzymes is based on a fluorogenic reaction within femtoliter chamber arrays. In this modality, the solution containing target enzyme and fluorogenic substrate was dropped onto the arrays and confined to the chambers by dropping an oil. The concentration of the target enzyme in the sample can be accurately quantified with high sensitivity by counting the number of positive chambers that exhibit a fluorescent signal resulting from the enzymatic reaction. This method has recently been applied to the highly sensitive detection of disease-related enzymes in blood^5,16,17^, opening up possibilities for early disease diagnosis. As a proof of concept of the first modality, the femtoliter chamber arrays prepared using FP-A and CYTOP were used for the direct detection of model enzymes commonly used in digital bioanalysis, i.e. horseradish peroxidase (HRP) and β-galactosidase (β-Gal)^2^ (Fig. 4A). The number of positive chambers increased linearly over a wide range of concentration of target enzymes, regardless of the fluoropolymer type (Fig. 4B,C). The number of positive chambers for β-Gal exceeded the theoretical values based on the Poisson distribution by more than 10 times, whereas HRP showed almost identical results. A possible reason for this discrepancy is that the non-specific binding of β-Gal to fluoropolymers is more likely than that of HRP, resulting in a higher number of positive chambers. The limit of detection (LoD) was nearly identical for HRP (~ 9.5 fM) and β-Gal (~ 0.3 fM) regardless of fluoropolymer type (Fig. 4C), suggesting that FP-A could be used as an alternative to CYTOP for femtoliter chamber arrays in digital bioanalysis.

**Figure 5:**
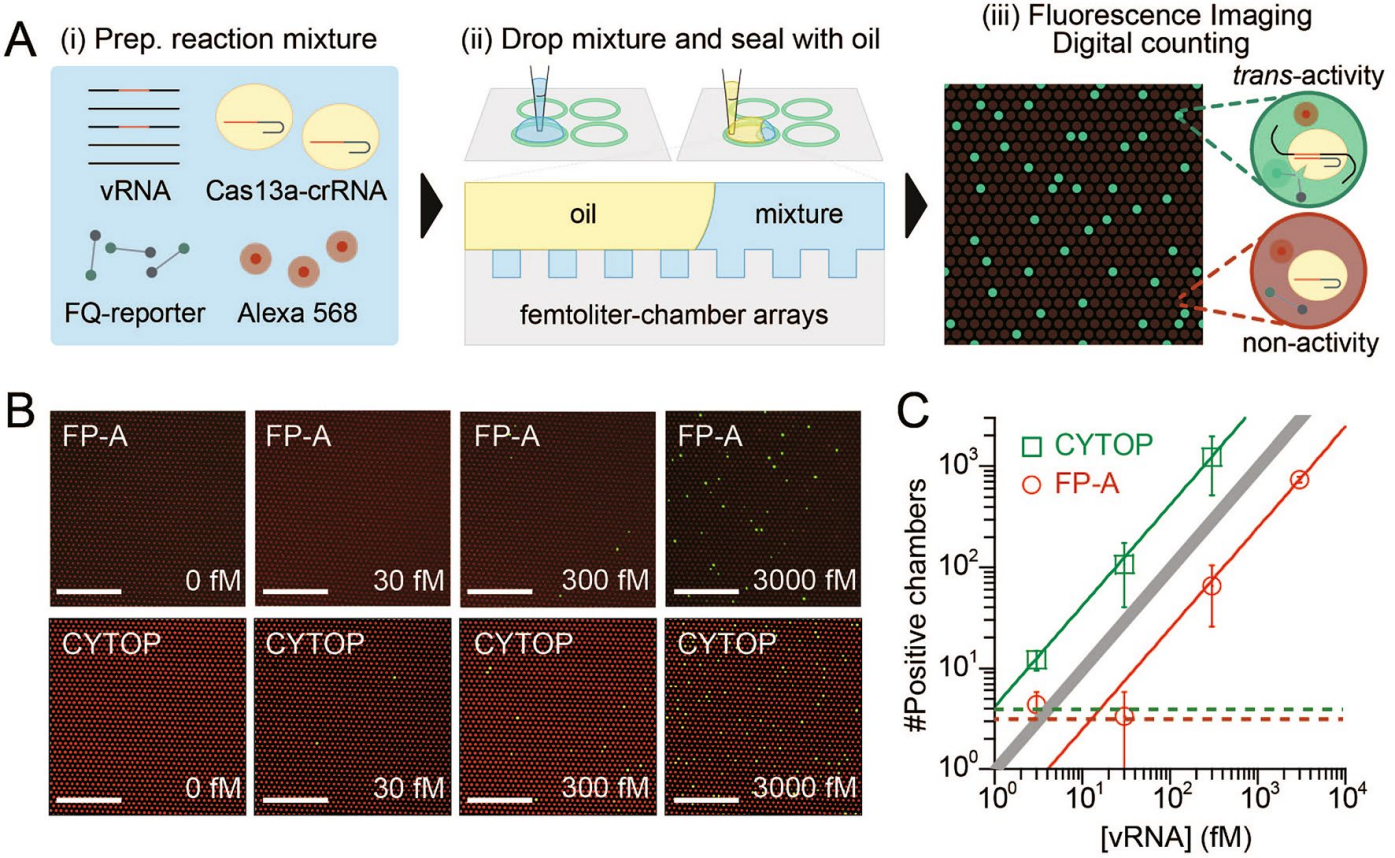
Amplification-free digital viral RNA detection (SATORI). (**A**) Schematics of SATORI assay. (**B**) Representative fluorescence images at each concentration of viral RNA using CYTOP and FP-A. Scale bars represent 100 µm. (**C**) Number of positive chambers obtained using CYTOP (green) or FP-A (red). The solid lines show the linear regressions in green and red and the theoretical value in grey. The dotted lines show the values of the blank mean + 3 SD (n = 3 technical replicates).

Indirect detection of target molecules by fluorogenic enzymes is another modality of digital bioanalysis. In this modality, fluorogenic enzymes are directly activated by one-to-one binding to target biomolecules, allowing indirect detection of target molecules from the fluorescent signal generated by the enzymatic reaction. Recently, nucleic acid cleavage enzymes such as CRISPR–Cas proteins, which are activated upon binding to specific nucleic acid sequences, have been widely adopted in biomedical applications^18,19^. Digital bioanalysis using these enzymes has attracted great attention in the diagnosis of infectious diseases, including COVID-19, as an innovative genetic testing method that bypasses the amplification process of genetic material ^11,12,20–23^. As a proof-of-concept of the second modality, digital viral RNA detection by CRISPR–Cas13a (SATORI)^20^ was performed using femtoliter chamber arrays prepared using FP-A, and the results were compared with those obtained using CYTOP (Fig. 5A). Considering the recent global pandemic, SARS-CoV-2 was selected as the target of the SATORI assay. In the SATORI assay, a mixture of viral RNA (vRNA), Cas13a complexed with crRNA (targeting the N-gene of SARS-CoV-2), and fluorescent reporters (FAM-based: FQ-reporter) was dropped onto the arrays and confined to the chambers by dropping an oil. After incubation for a few minutes, the chambers containing Cas13a–crRNA–vRNA complexes represented the fluorescence signal owing to the cleavage of the FQ reporters and were quantitatively counted as positive chambers from the fluorescence images obtained. This characteristic accelerates the quantification of viral RNA in samples, expediting digital bioanalysis for diagnosing viral infections compared to prior bulk assays that necessitated several tens of minutes of incubation to achieve an adequate fluorescence signal. The number of positive chambers increased linearly over a wide range of vRNA concentrations, regardless of the fluoropolymer type (Fig. 5B,C). However, the number of positive chambers for FP-A was approximately one tenth that for CYTOP, which was several times higher than the theoretical values based on the Poisson distribution. A possible reason for this discrepancy is that the non-specific binding of Cas13a to CYTOP is more likely than to FP-A, resulting in a higher number of positive chambers. The LoD for digital RNA detection was determined to be 13 fM and 0.96 fM for FP-A and CYTOP, respectively (Fig. 5C). The highly sensitive detection of viral RNA again demonstrated that FP-A could be used as an alternative for femtoliter chamber arrays in digital bioanalysis. Notably, the non-specific binding of target molecules leads to large variations in the number of positive chambers owing to slight perturbations in experimental procedures such as incubation time, indicating that FP-A may be suitable for quantitative digital bioanalysis.

## Discussion

We developed highly integrated femtoliter chamber arrays using various fluoropolymers commercially available. Among fluoropolymers tested, we found that FP-A is suitable for forming femtoliter chamber arrays. As a proof-of-concept, various digital bioanalyses were performed using the FP-A-based chamber array with model fluorogenic enzymes, including HRP, β-Gal, and Cas13a. The results demonstrated the similar performance to that of CYTOP with a LoD ranging from sub-fM to 10-fM, highlighting the broader utility of FP-A as an alternative to CYTOP in highly sensitive digital bioanalysis.

Femtoliter chamber arrays using PF-A have lower material costs than the conventional CYTOP; however, formation of thin films and the smooth performance of the photolithographic process remain a challenge. Therefore, it is necessary to develop low-cost and high-quality femtoliter chamber arrays by further optimizing the photolithographic conditions, as well as developing and exploring new materials with higher functionality. Collectively, these continuous efforts to improve femtoliter chamber arrays will pave the way for low-cost and robust digital bioanalysis, thereby contributing to the realization of more versatile diagnostics.

While environmental concerns are paramount, it's important to recognize supply chain challenges, including the availability of materials and reagent due to globalization. Delays in the supply of fluoropolymers and solvents from specific manufacturers, triggered by PFAS regulations^14^ primarily in Europe, highlight the critical need to establish a system capable of utilizing multiple materials from various manufacturers. This approach is essential to ensure the sustainability of various research and development efforts that will expand the versatility of digital bioanalysis and further provide fundamental knowledge for biomedical applications.

## Methods

### Fabrication of femtoliter chamber array

The through-hole structures of the fluoropolymers, used as a femtoliter chamber, were fabricated on a thin glass substrate using conventional photolithography^20^. The fabrication process comprised four steps: (i) spin coating of the fluoropolymer, (ii) spin coating of the photoresist, (iii) UV exposure and development, and (iv) dry etching.

A 32 mm × 24 mm cover glass (No. 1, Matsunami) was incubated overnight, sonicated for 1 h in 8 M KOH solution, rinsed with pure water, and dried using an air blow gun. For CYTOP (9% CYTOP, AGC), it was spin-coated on the glass at 1000 rpm, 4000 rpm, or 7000 rpm for 30 s, and then baked at 80 °C for 10 min and 180 °C for 1 h. Positive photoresist (AZ P4620, AZ Electronic Materials) was spin-coated on the CYTOP layer at 7500 rpm for 30 s, and then cured at 100 °C for 5 min. After rehydration of the photoresist at 25 °C for more than 5 min under 60% humidity, the glass was exposed to UV light, using a mask aligner (LA610dt, Nanometric Technology) and a chrome photomask with 1.5 μm holes, followed by incubation for 1.5 min in a developer (AZ300 MIF, AZ Electronic Materials). The area not covered by the photoresist was removed via dry etching with O_2_ plasma (RIE-10NR, Samco). Dry etching was performed at an O_2_ flow rate of 50 sccm, gas pressure of 10 Pa, RF power of 50 W, and etching time of 16 min. The fabrication of the femtoliter chamber array was completed by removing the remaining photoresist via sequential rinsing with acetone, 2-propanol, and pure water. The chamber volumes (8.8 fL, 4.2 fL, or 2.4 fL for 1000 rpm, 4000 rpm, or 7000 rpm, respectively) were determined using a laser microscope (VK-X1100, Keyence).

For FP-A (95% Fluorine Sealant DKA-001, DAIKIN INDUSTRIES), it was diluted 20 times with a fluorinated solvent (Novec 7200, 3 M), spin-coated on the glass at 1000 rpm, 4000 rpm, or 7000 rpm for 30 s, and then baked at 30 °C for 60 min and 70 °C for more than 12 h. Positive photoresist (AZ P4620, AZ Electronic Materials) mixed with 0.1% fluorosurfactant (Surflon S386, AGC seimi chemical) was spin-coated on the FP-A layer at 7500 rpm for 30 s. The temperature of the photoresist-coated glass was raised continuously from 40 to 100 °C using a hotplate, followed by curing for more than 5 min. After rehydration of the photoresist at 25 °C for 5 min under 60% humidity, the glass was exposed to UV light, using a mask aligner (LA610dt, Nanometric Technology) and a chrome photomask with 1.5 μm holes, and then incubated for 0.75 min in a developer (AZ300 MIF, AZ Electronic Materials). The area not covered by the photoresist was removed via dry etching with O_2_ plasma (RIE-10NR, Samco). Dry etching was performed at an O_2_ flow rate of 150 sccm, gas pressure of 10 Pa, RF power of 80 W, and etching time of 15 min. The remaining photoresist was removed via sequential rinsing with acetone, 2-propanol, and pure water. The fabrication of the femtoliter chamber array was completed by sonication with pure water for 5 min and with 4 M KOH solution for 10 min, followed by rinsing with pure water, and drying using an air blow gun. The chamber volumes were 5.1, 2.8, or 3.8 fL for 1000, 4000, or 7000 rpm, respectively.

For FP-B (13% SFCOAT SFE-GP03JL AGC seimi chemical), it was diluted 1.5 times with a fluorinated solvent (Ct-Solv180, AGC), spin-coated on the glass at 1000 rpm, 4000 rpm, or 7000 rpm for 30 s, and then baked at 80 °C for 10 min and 180 °C for 1 h. Positive photoresist (AZ P4620, AZ Electronic Materials) was spin-coated on the FP-B layer at 7500 rpm for 30 s, and then cured at 100 °C for 5 min. After rehydration of the photoresist at 25 °C for 5 min under 60% humidity, the glass was exposed to UV light, using a mask aligner (LA610dt, Nanometric Technology) and a chrome photomask with 1.5 μm holes, and then incubated for 1.5 min in a developer (AZ300 MIF, AZ Electronic Materials). The area not covered by the photoresist was removed via dry etching with O_2_ plasma (RIE-10NR, Samco). Dry etching was performed with an O_2_ flow rate of 50 sccm, gas pressure of 10 Pa, RF power of 50 W, and etching time of 3 min at a spin speed of 1000 rpm, and 5 min at a spin speed of 4000 rpm and 7000 rpm. The fabrication of the femtoliter chamber array was completed by removing the remaining photoresist via sequential rinsing with acetone, 2-propanol, and pure water. The chamber volumes were 6.7 fL or 5.4 fL at 4000 rpm, or 7000 rpm, respectively.

For FP-C (0.1% SURECO 2101S, AGC), the film thickness was not enough to be analyzed with the laser microscope, regardless of the spin speed. For FP-D (20% SURECO 2120X, AGC), the thin film collapsed and droplets formed when it was baked on a hotplate.

After the photolithography process, 7-mm ring-shaped enclosures were fabricated as a reaction well on the substrate using a UV-curable acrylic resin (5X649H, CHEMITECH) and a dispensing robot (SHOTmini 200SX SM200S, Musashi Engineering).

### Acquisition of scanning electron microscope (SEM) images

A thin platinum layer was formed on the surface of the femtoliter chamber array via sputtering (JEC-3000FC, JEOL). High magnification and resolution images of the femtoliter chamber array were captured using a scanning electron microscope (JSM-IT210, JEOL) with 20 kV acceleration in a high vacuum environment.

### Fluorescent dye encapsulation assay

The fluorescent dye solutions were prepared by dissolving indicated amount of Alexa Fluor™ 488 C 5 Maleimide (Alexa 488) and Alexa Fluor™ 568 C 5 Maleimide (Alexa 568) in buffer A (20 mM HEPES–KOH [pH 6.8], 100 mM KCl, 10 mM MgCl_2_, and 50 µM Triton X-100). The 105 μL of the fluorescent dye solution was dropped onto a femtoliter chamber array. Then, 95 μL was removed, and 50 μL of Fomblin oil (Y LVAC 25/6, Solvay) was added to seal the chamber. The excess solution and Fomblin oil left on the array were removed. After 1 min of incubation, fluorescence images (10-bit TIFF) were acquired with 488 nm (green) and 561 nm lasers (orange) using confocal microscope (A1HD25, Nikon) with a 20 × objective lens (NA = 0.75). There were more than 12,000 femtoliter chambers in one field of view.

The fluorescence images were analyzed using the ImageJ software (National Institute of Health), as follows: The orange images were processed using the ImageJ setThreshold command (parameters: 400–65,535 for CYTOP, 1200–65,535 for FP-A, and 200–65,535 for FP-B), and Analyze Particles command (parameters: size = 10–200, circularity = 0.30–1.00) to determine the location of femtoliter chambers. The ROI of the orange images were used for the green images in the same field of view, which were then processed using the roiManager command (parameter: Measure) to measure the fluorescence intensity of Alexa 488 in the chambers.

### Fluorescence after photobleaching (FRAP)

The femtoliter chambers were filled with buffer A containing 10 µM Alexa 488 and 2 µM Alexa 568 and then sealed with Fomblin oil. After 3 min of fluorescence imaging, the central rectangular area was selectively photobleached for approximately 50 s using the laser scanning system of the confocal microscope. Fluorescence images were acquired at intervals of 60 s over 20 min.

### Autofluorescence measurement

Autofluorescence images of femtoliter chamber arrays were acquired using a confocal microscope at four excitation wavelengths (405, 488, 561, and 640 nm). The ratios of the autofluorescence intensities of FP-A and FP-B to that of CYTOP were then calculated.

### Preparation of Cas13a

For the expression of LtrCas13a, *Escherichia coli* Rosetta 2 (DE3) was transformed with the pET-LtrCas13a plasmid, and the cells were cultured in a 2.5 L of LB medium containing kanamycin. When the OD_600_ values reached 0.6–1.0, the cells were cooled on ice for 10 min and further cultured at 20 °C for 20 h with 0.1 mM IPTG. Bacterial cells were collected by centrifugation, suspended in 40 mL of buffer B (20 mM Tris–HCl [pH 8.0], 1 M NaCl, 20 mM imidazole, 3 mM β-mercaptoethanol, and 1 mM phenylmethylsulfonyl fluoride), and lysed via sonication (Q500, QSONICA). After centrifugation at 15,000 rpm for 20 min, the supernatant was incubated with Ni–NTA agarose (Qiagen) at 4 °C for 1 h. The mixture was then transferred to an Econo column (Bio-Rad). The resin was washed with buffer C (20 mM Tris–HCl [pH 8.0], 0.3 M NaCl, 20 mM imidazole, and 3 mM β-mercaptoethanol), and the protein was eluted with buffer D (20 mM Tris–HCl [pH 8.0], 0.3 M NaCl, 300 mM imidazole, and 3 mM β-mercaptoethanol). The protein was then loaded onto a HiTrap SP HP column (Cytiva) equilibrated with buffer E (50 mM HEPES–KOH [pH 7.5], 0.3 M NaCl, and 0.5 mM TCEP). The protein was eluted using a linear gradient from 0.3 to 2.0 M NaCl over seven column volumes. It was further purified through size exclusion chromatography (Enrich SEC 650, Bio-Rad) with buffer F (50 mM HEPES–KOH [pH 7.5], 0.5 M NaCl, and 0.5 mM TCEP).

### Preparation of crRNA, FQ-reporter, and viral RNA

Chemically synthesized crRNAs targeting the N-gene of SARS-CoV-2 (GGAUUUAGAGUACCCCAAAAAUGAAGGGGACUAAAACAAGGUCUUCCUUGCCAUGUUGAGUGAGAGCGG) were purchased from GeneDesign or Hokkaido System Science. The FQ-reporter (FAM/rUrUrUrUrU/3lABkFQ) were purchased from IDT. The viral RNA fragment (N-gene, SARS-CoV-2) was transcribed in vitro with T7 RNA polymerase at 37 °C for 1 h, using a partially double-stranded DNA template. To remove the double-stranded RNA contaminations, the transcribed RNA was incubated with RNaseIII (New England Biolabs) at 37 °C for 30 min, and then purified by 8% native polyacrylamide gel electrophoresis. The RNA concentration was determined from the A_260_ value measured by the NanoDrop spectrophotometer.

### SATORI assay

The assay solution for SATORI (solution A) for a single assay was prepared as follows: To prepare Cas13a-crRNA complexes, a mixture of 0.7 μL of Cas13a (20 μM), 1.4 μL of crRNA, and 2.6 μL of buffer G (20 mM HEPES–KOH [pH 6.8], 60 mM NaCl, 6 mM MgCl_2_, and 50 μM Triton X-100) was incubated at 37 °C for 10 min. Next, 4.7 μL of Cas13a-crRNA solution was mixed with 18.7 μL of buffer H (20 mM HEPES–KOH [pH 7.5], 100 mM NaCl, 10 mM MgCl_2_, 50 μM Triton X-100, 15 μM FQ-reporter, and 15 μM Alexa 568) and stored at − 80 °C until use.

Before starting the SATORI assay, frozen solution A was thawed at 25 °C. Then 20 μL of solution A was mixed with 100 μL of target RNA solution. After 1 min of incubation, 105 μL of the mixture was dropped onto an femtoliter chamber array. Of the 105-μL solution on the array, 95 μL was removed, and 50 μL of Fomblin oil was added to seal the chamber. The excess solution A and Fomblin oil remaining on the array were removed. After 5 min of incubation, fluorescence tiling images were acquired using a confocal microscope (A1HD25, Nikon) with 25-stage scanning, acquired with 488 nm (green) and 561 nm lasers (red) with a 20 × objective lens (NA = 0.75).

### Data analysis for SATORI assay

The fluorescence images were analyzed using the ImageJ software as follows: the green images were processed using the ImageJ setThreshold command (parameters: 200–65,535), Analyze Particles command (parameters: size = 200–Infinity, circularity = 0.00–1.00), and roiManager (parameter: Fill) to remove large defective areas originating from the liquid sumps. The green images without defective areas were further processed using the setThreshold command (parameters: 1500–65,535) and Analyze Particles command (parameters: size = 15–Infinity, circularity = 0.40–1.00) to determine the positive chambers.

The analytical limit of detection (LoD) was defined as follows: the number of positive chambers obtained with different concentrations of the viral RNA fragment (vRNA) was fitted to a linear curve. The mean + 3 SD value for the number of positive chambers obtained without vRNA was determined, and the crossing point of the linear curve and the mean + 3 SD value were then determined. The concentration corresponding to the crossing point was defined as the LoD value.

### Preparation of HRP, β-Gal, and their fluorogenic substrates

HRP was purchased from Fujifilm Wako Chemicals (# 169-10791). HRP concentration was determined by BCA method using the molecular weight of HRP as 40.2 kDa. Amplex Red, a fluorogenic substrate for HRP, was purchased from Invitrogen (# A12222). β-Gal was purchased from MP Biomedicals (#104939). β-Gal concentration was determined by BCA method, using a molecular weight of tetrameric β-Gal as 540 kDa. Resorufin-β-D-galactopyranoside (RGP), a fluorogenic substrate for β-Gal, was purchased from sigma Aldrich (# R4883).

### Digital enzyme assay

For digital HRP assay, 20 μL of solution B (600 µM Amplex Red, 30 µM Alexa 647, 6 mM H_2_O_2_, 20 mM HEPES–KOH [pH 7.5], 100 mM NaCl, 10 mM MgCl_2_, and 50 μM Triton X-100) was mixed with 100 μL of solution C (Arbitrary concentration of HRP, 20 mM HEPES–KOH [pH 7.5], 100 mM NaCl, 10 mM MgCl_2_, and 50 μM Triton X-100). Just after mixing these two solutions, 105 μL of the mixture was dropped onto an femtoliter chamber array. Of the 105-μL solution on the array, 95 μL was removed, and 50 μL of Fomblin oil was added to seal the chamber. The excess solution and Fomblin oil remaining on the array were removed. After 8 min of incubation, fluorescence tiling images were acquired using a confocal microscope (A1HD25, Nikon) with 25-stage scanning, acquired with 561 nm (green) and 640 nm lasers (red) with a 20 × objective lens (NA = 0.75).

For digital β-Gal assay, 20 μL of solution D (600 µM RGP, 30 µM Alexa 647, 20 mM HEPES–KOH [pH 7.5], 100 mM NaCl, 10 mM MgCl_2_, and 50 μM Triton X-100) was mixed with 100 μL of solution E (Arbitrary concentration of β-Gal, 20 mM HEPES–KOH [pH 7.5], 100 mM NaCl, 10 mM MgCl_2_, and 50 μM Triton X-100). Just after mixing these two solutions, 105 μL of the mixture was dropped onto an femtoliter chamber array. Of the 105-μL solution on the array, 95 μL was removed, and 50 μL of Fomblin oil was added to seal the chamber. The excess solution and Fomblin oil remaining on the array were removed. After 4 min of incubation, fluorescence tiling images were acquired using a confocal microscope (A1HD25, Nikon) with 25-stage scanning, acquired with 561 nm (green) and 640 nm lasers (red) with a 20 × objective lens (NA = 0.75).

### Data analysis for digital enzyme assay

The fluorescence images were analyzed using the ImageJ software as follows: the green images were processed using the ImageJ setThreshold command (parameters: 600–65535), Analyze Particles command (parameters: size = 200–Infinity, circularity = 0.00–1.00), and roiManager (parameter: Fill) to remove large defective areas originating from the liquid sumps. The green images without defective areas were further processed using the setThreshold command (parameters: 900–65535) and Analyze Particles command (parameters: size = 15–40, circularity = 0.50–1.00) to determine the positive chambers. The analytical limit of detection (LoD) was defined as follows: the number of positive chambers obtained with different concentrations of the target enzymes was fitted to a linear curve. The mean + 3 SD value for the number of positive chambers obtained without target enzymes was determined, and the crossing point of the linear curve and the mean + 3 SD value were then determined. The concentration corresponding to the crossing point was defined as the LoD value.

### Supplementary Information

Supplementary Figures.

## Data Availability

All data needed to evaluate the conclusions in the paper are provided in the paper and/or the Supplementary Materials.
