# Supplementary material for: Exploring fluoropolymers for fabrication of femtoliter chamber arrays used in digital bioanalysis
Source: Sci Rep. 2024 May 20;14:11442. doi: 10.1038/s41598-024-61726-8 (PMC11106263; doi:10.1038/s41598-024-61726-8)
Supplement: Supplementary file 1 — Supplementary Figures. [file 41598_2024_61726_MOESM1_ESM.pdf]

Supporting Information

Exploring fluoropolymers for fabrication of femtoliter chamber arrays used in digital bioanalysis

Jun Ando, Kazue Murai, Makiko Mori, Tomoe Michiyuki, Tatsuya Iida, Asami Makino, Hajime Shinoda, and Rikiya Watanabe

Contents

Figures S1–S6

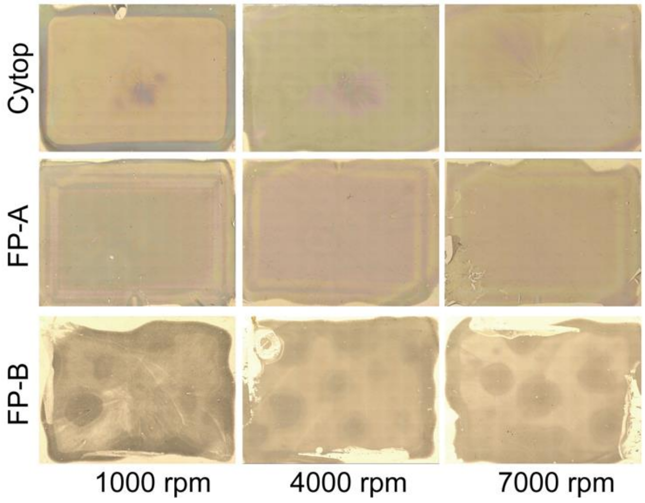

Fig. S1 Photographs of femtoliter chamber arrays fabricated using fluoropolymers, CYTOP, FP-A and FP-B. The fluoropolymer film was formed by spin-coating at 1,000 rpm, 4,000 rpm and 7,000 rpm, respectively.

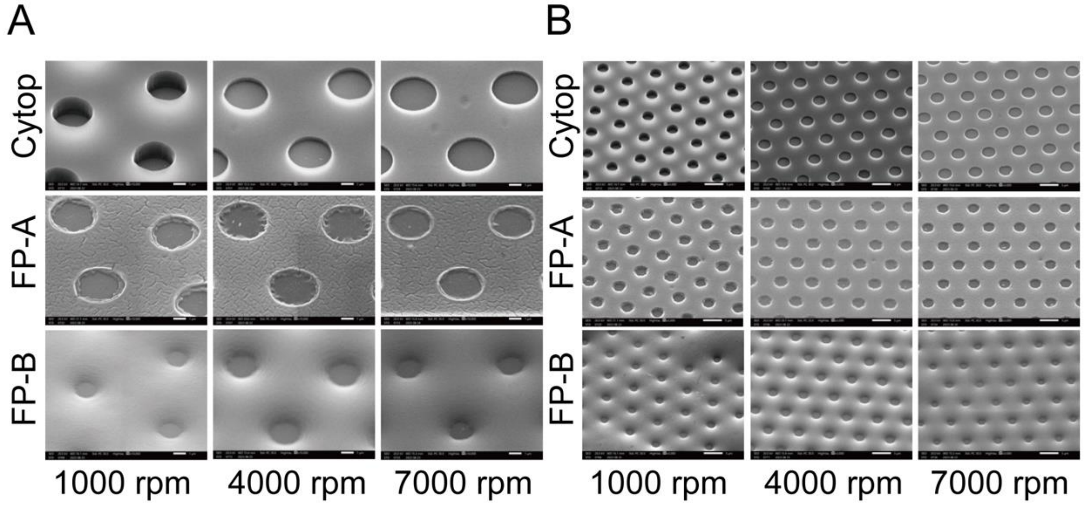

Fig. S2 SEM images of femtoliter chambers fabricated using CYTOP, FP-A and FP-B. Magnifications are (A) 10,000x and (B) 3,000x.

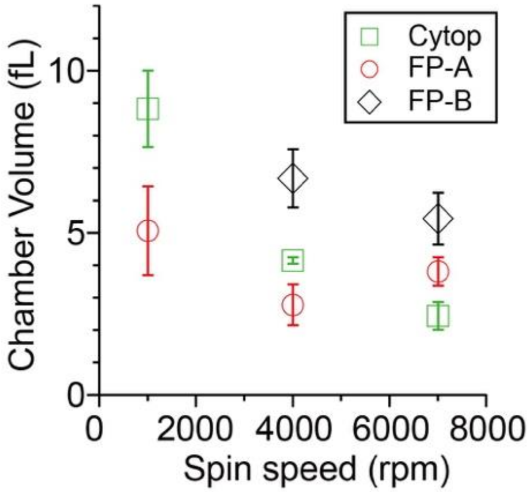

Fig. S3 Chamber volume of femtoliter chambers fabricated using CYTOP, FP-A and FP-B (n = 3 replicates).

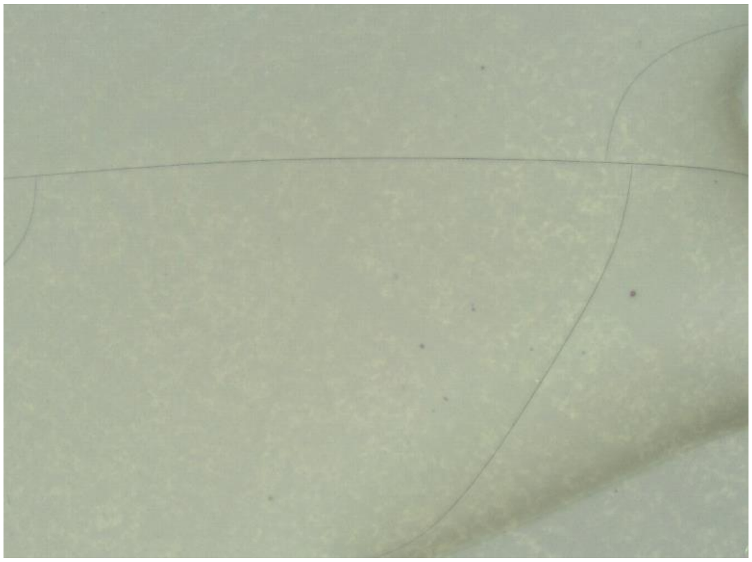

Fig. S4 Photographs of cracks in the deposited photoresist film on top of FP-A after vacuum condition in a RIE machine.

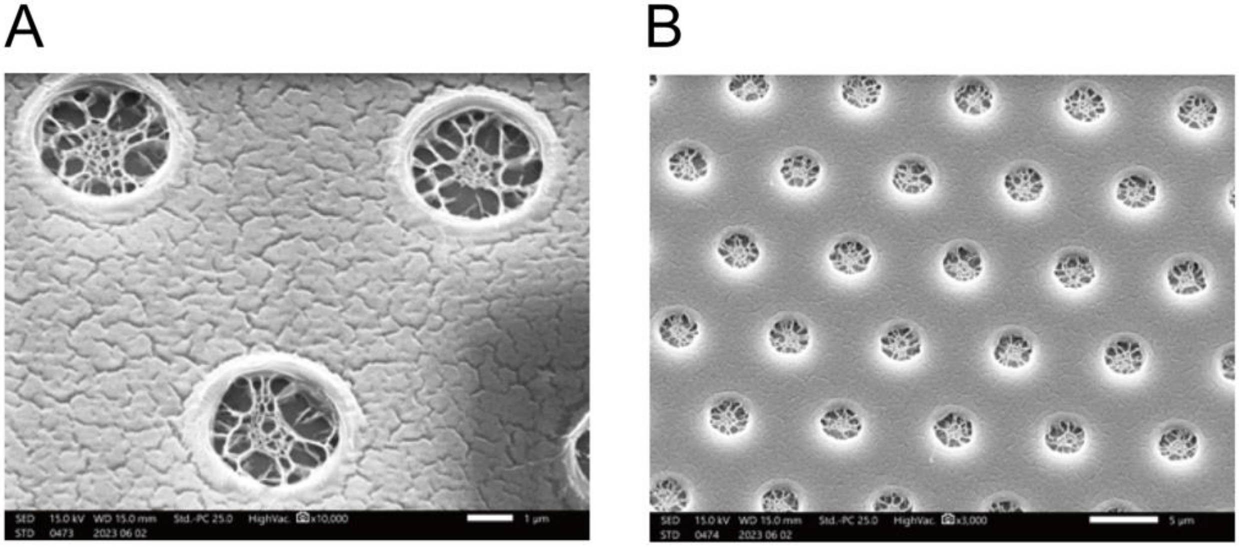

Fig. S5 SEM images of mesh structures remained in femtoliter chambers fabricated with FP-A after dry etching. Magnifications are (A) 10,000x and (B) 3,000x.

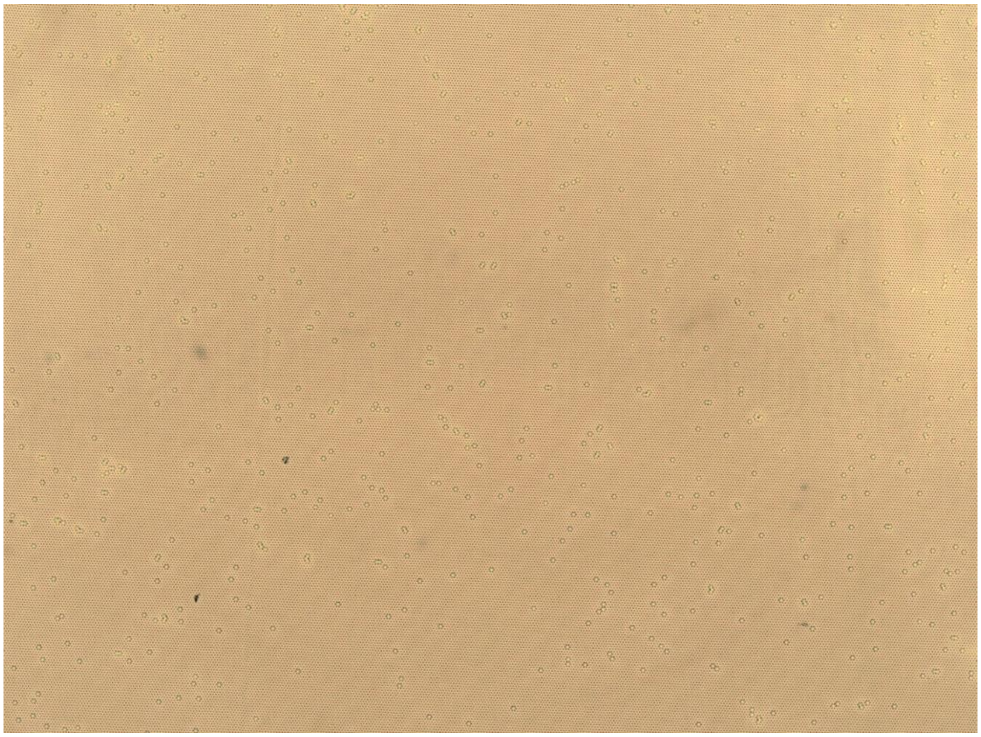

Fig. S6 Photograph of protrusions in femtoliter chamber arrays fabricated with FP-B
